# NoDe: a fast error-correction algorithm for pyrosequencing amplicon reads

**DOI:** 10.1186/s12859-015-0520-5

**Published:** 2015-03-15

**Authors:** Mohamed Mysara, Natalie Leys, Jeroen Raes, Pieter Monsieurs

**Affiliations:** Unit of Microbiology, Belgian Nuclear Research Centre (SCK CEN), Mol, Belgium; Department of Bioscience Engineering, Vrije Universiteit Brussel, Brussels, Belgium; VIB Center for the Biology of Disease, VIB, Leuven, Belgium; Department of Microbiology and Immunology, REGA institute, KU, Leuven, Belgium

**Keywords:** Error correction, Denoising, 16S rRNA amplicon sequencing, 454 pyrosequencing, Metagenomics

## Abstract

**Background:**

The popularity of new sequencing technologies has led to an explosion of possible applications, including new approaches in biodiversity studies. However each of these sequencing technologies suffers from sequencing errors originating from different factors. For 16S rRNA metagenomics studies, the 454 pyrosequencing technology is one of the most frequently used platforms, but sequencing errors still lead to important data analysis issues (e.g. in clustering in taxonomic units and biodiversity estimation). Moreover, retaining a higher portion of the sequencing data by preserving as much of the read length as possible while maintaining the error rate within an acceptable range, will have important consequences at the level of taxonomic precision.

**Results:**

The new error correction algorithm proposed in this work - NoDe (Noise Detector) - is trained to identify those positions in 454 sequencing reads that are likely to have an error, and subsequently clusters those error-prone reads with correct reads resulting in error-free representative read. A benchmarking study with other denoising algorithms shows that NoDe can detect up to 75% more errors in a large scale mock community dataset, and this with a low computational cost compared to the second best algorithm considered in this study. The positive effect of NoDe in 16S rRNA studies was confirmed by the beneficial effect on the precision of the clustering of pyrosequencing reads in operational taxonomic units.

**Conclusions:**

NoDe was shown to be a computational efficient denoising algorithm for pyrosequencing reads, producing the lowest error rates in an extensive benchmarking study with other denoising algorithms.

**Electronic supplementary material:**

The online version of this article (doi:10.1186/s12859-015-0520-5) contains supplementary material, which is available to authorized users.

## Background

The introduction of next generation sequencing technologies has led to important breakthroughs throughout the life sciences, with applications in de novo genome, exome or amplicon sequencing, gene expression analysis, identification of transcription factor binding sites, and so on. Also in clinical and environmental microbial community analysis, 16S rDNA sequencing and metagenomics have been instrumental. For the assessment of microbial community structures based upon 16S rDNA amplicon sequencing, the 454 pyrosequencing platform has already been in use for many years, mainly due to its longer read length [1] nowadays allowing up to 1000 bp reads.

Having access to highly reliable sequencing data is a necessary requirement for biodiversity assessments using 16S rRNA amplicons [2,3] as this approach is highly sensitive to sequencing errors. Indeed, the natural variation in the 16S marker genes for different bacterial species significantly complicates the problem of distinguishing between erroneous sequences on the one side, and sequencing reads representing rare taxa on the other side. This may lead to an overestimation of the number of operational taxonomic units (OTUs) and sample biodiversity as a whole [1,3,4]. Therefore, error-correction prior to starting biological interpretation of the data is a matter of utmost importance.

Several efforts have been made to study the sources of those errors and how to eliminate or correct them [5-8]. There are three major causes of errors in sequencing data at different stages in the sequencing process: i) errors originating from usage of the PCR polymerase enzymes (with error rate of 0.000,010 to 0.000,001 per nucleotide [9]), ii) PCR artifacts, known as chimeras [10], and iii) errors originating from the sequencing platform [5]. Concerning the latter type of errors, different indicators have already been identified for the GS20 [11] and GS-FLX Titanium [5,8,12] platforms: (i) position within the read i.e. the quality of the read is dropping with increasing position in the read, (ii) the presence of homopolymers gives difficulties to identify the correct length of the homopolymer or cause an insertion or substitution near it (i.e. carry-forward events), (iii) abnormality in the read length (i.e. suspiciously short or long) that may be caused by extreme quality filtering, accumulation of errors or stochastic polymerization ending, (iv) position of the bead on the plate (edge effect), (v) distribution of the errors, as errors tend to accumulate in small subset of sequencing reads, meaning that a majority of reads will be error-free or only contain a single position error while a minority of reads is problematic, (vi) nucleotide type, as mismatch transitions are not of equal rates.

Different methods have been developed to enhance the quality of pyrosequencing reads, starting with the most basic approaches e.g. by removing those reads where no perfect match with the PCR primer could be identified [13]. Another approach for correcting sequencing errors was introduced by Huse and co-workers [11], including: i) removal of reads with one or more ambiguous bases, ii) reads with a length outside the main distribution or iii) reads containing mismatches in their primer sequence. Additionally, a major improvement was achieved via the use of the read quality scores (i.e. the Phred scores) as proposed in Kunin et al. [4] by trimming reads from the most upstream position where the quality dropped below the assigned cut-off quality score. Similarly, Schloss et al. [5] implemented a method where trimming of sequencing reads was based on a drop in the average quality of the read in total or within a specific sliding window.

In addition to methodologies trimming or removing sequencing reads, more sophisticated approaches were made by clustering of the 454 standard flowgrams, to better handle homopolymer related errors, either by applying an expectation maximization (EM) algorithm as in PyroNoise [6] or greedy scheme as implemented in Denoiser [14]. Three sequence based clustering algorithm with a much faster computation time were developed quite recently, namely the Single Linkage Pre-clustering method (SLP) [15], AmpliconNoise (including an update version of PyroNoise and SeqNoise) [16] and Acacia [17]. SLP clusters low redundant reads containing errors with the more redundant error-free ones. This is clustering is based on the pairwise distance scores between both reads, thereby tolerating some errors (aiming not to cross the interspecies threshold). AmpliconNoise consists of two steps: i) the PyroNoise algorithm, which applies a clustering approach directly on the flowgrams rather than relying on the quality scores assigned by the 454 pyrosequencing platform, and ii) SeqNoise, which applies a sequence clustering step.

Acacia [17] is denoising algorithm that aligns each read to dynamically updated cluster consensus sequences, thereby avoiding the time-consuming all-against-all alignments, and is mainly focusing on correcting sequencing errors in homopolymer regions. SLP was re-implemented in mothur [18] as the pre.cluster command. In contrast to SLP, pre.cluster is applied on the aligned sequences thereby avoiding the computationally intensive all-against-all alignments. Pre-cluster was found to outperform SLP in terms of speed and performance as it avoids overclustering of sequences (i.e. described as the “chaining effect” of SLP [5,19]) since Pre-cluster only groups reads when they are within a maximum distance to the cluster center. These implementations make use of the sequence frequencies to remove errors based on the relative abundance of an error versus a correct nucleotide. Since SLP and Pre-cluster both work directly on the sequencing data (and not on the flowgrams), those algorithms do not distinguish between PCR point errors and errors produced by the sequencing technology.

When dealing with error-correcting algorithms, researchers need to find a balance between the quantity (in terms of the number of sequences and their average length), and the quality of their pyrosequencing reads (in terms of the error rates i.e. rates of deletions, insertions or substitutions). Retaining more sequencing data by increasing the average read length, while keeping the error rate within an acceptable range, will have important consequences for taxonomic precision. Another factor that needs to be taken into consideration is the computational cost, which is getting more important with the dramatic increase of the size of sequencing data, resulting in a need for fast and accurate methods to analyse them. Here, we introduce a novel way of artificial intelligence-based prediction of erroneous positions in sequencing reads (using a support vector machine trained classifier) and subsequent clustering which will correct error-containing reads in the sequencing data by grouping them with error-free reads (using a modified version of the SLP algorithm), thereby fulfilling the need for quality and speed. This methodology was benchmarked against other state-of-the-art algorithms and multi-step methodologies.

## Methods

### Mock communities

In this work, previously published mock datasets were used, as well as a new in-house made mock community. First we used the mock datasets presented in Schloss et al. [5] as available online (Project “SRP002397” in NCBI Short Read Archive) consisting of 69 samples (21 samples missing in the Short Read Archive), targeting three hypervariable regions in the 16S rRNA gene i.e. V1 to V3, V3 to V5 and V6 to V9 (total of 888,635 reads. Due to missing data as described above, the sff-files were reconstructed (information provided by P. Schloss, personal communication), and are made available on the NoDe website. One of these samples was randomly selected for training the classifier (accession number F01QS4Z01_rep1_v35, 13,598 reads) and the other 68 samples were used for testing (referred to as TrainingDB and MOCK1 respectively in the remainder of this work). Although the composition of MOCK1 samples was known (20 bacterial species belonging to 18 families), we did not have access to the exact concentrations for each species.

The in-house built mock community (called MOCK2), consists of 17 bacterial species, namely *Acidovorax defluvii, Pseudomonas xanthomarina, Pseudomonas aeruginosa, Paracoccus denitrificans, Rhodospirillum rubrum, Microbacterium phyllosphaerae, Arthrobacter oryzae, Delftia tsuruhatensis, Nitrosomonas europaea, Cupriavidus metallidurans, Clostridium botulinum, Staphylococcus aureus, Bacillus cereus, Arthrospira platensis, Enterococcus faecium, Yersinia enterocolitica,* and *Desulfovibrio oxamicus* ordered by their concentrations in descending order.

The DNA was extracted from the individual cultures, mixed, and PCR amplified (30 cycles). Next, the DNA mixture was sequenced in triplicate using the 454 GS-FLX Titanium sequencing platform, covering the region V1-V3 (primer pair AGAGTTTGATCCTGGCTCAG and TTACCGCGGCTGCTGGCAC. Creating an uneven mock community (i.e. all organisms are present in different relative abundances within a theoretical range between 0.5% and 50%) allowed us to test the capability of each tool and pipeline to recover the exact initial microbial composition. To have absolute confidence in the reference genomes used for calculating the error rates, the exact 16S rRNA gene sequence for the 17 species was obtained using Sanger sequencing. For all paralogous 16S rRNA genes within the mock community, no differences between paralogs could be observed for 13 species. For the 4 species with sequence variations between the paralogs (all in species present in lower than 1% in the uneven mock community), only one difference (for eight paralogs) or three differences (for two paralogs) could be observed. However, this variation could not contribute more than 0.1% to the total error rate, and will certainly not lead to an inflation of OTUs in the downstream analysis as the percentage difference is much lower than the 3% difference cut-off used. As we have to take into account the technical (e.g. pipetting errors) and PCR bias that might result in an aberration from the presumed concentrations, the raw sequencing reads were mapped back using NAST [20] to the 17 reference genomes, leading to a compositional range between 0.30% and 55.8% (see Additional file 1). The sequencing data are submitted to the NCBI Sequence Read Archive (PRJNA257992). Applying basic trimming (described in detail in the “preprocessing sequencing data” section) resulted in a mock community data set consisting of 145,245 reads with an average length of 480 and a raw error rate of 0.0031.

### Error calculation and chimera detection

To obtain erroneous positions (i.e. “the ground truth”) in the TrainingDB dataset, each read was BLASTed [21] against a database containing all the reference sequences of the corresponding mock community. After finding the potential reference sequence, an accurate alignment was produced using ClustalW [22] adjusting the parameters as recommended in Gilles et al. [12], to get the highest accuracy for the identification of insertions, deletions and mismatches. This computationally costly approach was needed to get the highest sensitivity in identifying those errors that are used as training data to build the classifier, and to obtain the positional information of the sequencing errors needed to train the classifier.

In the comparative study between different tools, the global error rate was used to evaluate the accuracy of each approach, reflecting the number of erroneous nucleotides over the total number of nucleotides. For this benchmarking step the mothur command seq.error as implemented in Schloss et al. [5] was used to calculate the over-all error rate. Importantly, this algorithm also implements a highly efficient approach to detect chimeric sequences in mock communities. In a first step, both the reference sequences and sequencing reads were aligned using the NAST algorithm to the SILVA reference alignment. In the second step, chimera were detected via calculating the number of mismatches between each 454 pyrosequencing read and all possible two-parent chimera that could be generated by the reference sequences. In those cases where a sequence is at least three bases more similar to a multi-reference chimera than to a single reference sequence it is considered a chimera, and thus excluded from error calculations. The percentage of chimera was found to be 8.6% and 2.0% for MOCK1 and MOCK2 reads. Additionally to the chimera detection step, the error rate is calculated via counting the distance between the 454 reads and closest reference sequence. It was applied on both MOCK1 and MOCK2 for the non-denoised data and after each different denoising tool.

### Denoising algorithms

Our newly introduced denoising algorithm was benchmarked with four other commonly used denoising algorithms: single linkage preclustering (SLP) [15], AmpliconNoise [16], Acacia [17] and Denoiser [14]. For SLP we used the implementation pre.cluster as available in mothur [18] (version 1.33.3). We will refer to this algorithm as Pre-cluster in the text below. Similarly, AmpliconNoise (PyroNoise and SeqNoise) was run using the mothur commands shhh.flow and shhh.seqs. For Denoiser we used Mac-QIIME implementation via the denoise_wrapper.py (version 1.5.0) script. For Acacia we used the original implementation (version 1.52) as available online. All algorithms were run using their default parameters.

### Preprocessing sequencing data

As shown in the introduction, trimming of pyrosequencing reads is a common preprocessing step in 16S rRNA amplicon sequencing [4,5,11]. In order to allow a fair comparison between different denoising algorithms, the same input data should be used for all tools, thereby preventing the confounding effect of using different pre-processing pipelines. Therefore, the same basic preprocessing approach was applied as proposed in Schloss et al. [5], i.e. culling reads with one or more ambiguities, removing too short reads (<200 bp), and filtering out reads with homo-polymers longer than 8 bp. This approach – what we will refer to as “basic trimming” – is applied on all datasets discussed in this work.

Following the basic trimming step, optionally a more stringent trimming approach – further referred to in the text as “strict trimming” – can be applied. For preprocessing the data used as input for Pre-cluster, Acacia, Denoiser and our newly developed approach (NoDe), a sliding window approach was used to trim reads until the position where the average quality of this window drops below a cut-off Phred score (trim.seqs command in mothur [18]). As mentioned in the introduction, the aim of this work is to develop a denoising algorithm resulting in an acceptable error rate, while preserving longer read lengths. These longer read lengths can be guaranteed by using a sliding window of 100 nt and a cut-off on the average Phred score of 30. For AmpliconNoise, a similar effect could be achieved by trimming the sequencing data according to the guidelines stated in the original paper describing the tool, [16], by retaining only those reads with minimum flow length of 360 and maximum flow length of 720.

For both approaches (basic and strict trimming) reads were aligned to a 50,000-column wide SILVA-based reference alignment [23], using a NAST-based aligner [20], as available in mothur [24] and filtered (align.seqs and filter.seqs commands in mothur respectively), and subsequently subjected to error calculation (using seq.error) for comparative analysis. For assessing the impact of the error correcting algorithms on the OTU clustering, reads were clustered using the clustering algorithm as integrated in UPARSE (a 0.97 cut-off without singletons removal) using the UPARSE command with the following options: sortbysize, cluster_otus, and usearch_global [25]. Next, reads were classified using the RDP classifier [26] with 80% cutoff by applying mothur classify.seqs command. First, we applied this clustering approach (i.e. clustering with cut-off 0.03 and classified using the RDP classifier) on the selected V1-V3 region of the correct reference sequences to assure that a correct taxonomic classification could be obtained theoretically (i.e. by working on the correct reference sequences) (see Additional file 2). The same algorithmic approach was used for taxonomic classification of the MOCK2 reads after applying the pre-processing as mentioned above.

## Results

### NoDe (Noise Detector) algorithm

Our algorithm consists of two steps. First, a pre-trained classifier is used to identify those positions in the reads that are conceivable to be erroneous nucleotides based on a list of features potentially acting as a predictor for sequencing errors. In a second step the SLP algorithm [15] as implemented in mothur [18] is adapted in such a way that those nucleotides being marked as potentially erroneous are not penalized in the mismatch counting used to cluster similar reads. Both steps are explained more in detail below.

For training the NoDe classifier, an exhaustive list of features potentially able to predict sequencing errors was derived based on conclusions presented in Schloss et al. [5], Gilles et al. [12] and Huse et al. [15]: i) the position in the read, ii) PhreD score, iii) the presence and exact location within a homopolymer, iv), the possibility whether this position is sensitive to carry forward events, and v) the flowgram signal intensity. Additionally we also examined the predictive effect of characteristics derived from neighbouring nucleotides (one position before and after the investigated nucleotide): i) the Phred Score, ii) the presence and exact location within a homopolymer, iii) flowgram signal intensity and iv) the highest flowgram signal intensity score that did not result in a base call, measured over all signal intensity levels in the flowgram between the position studied and the neighboring nucleotide. Combining those features lead to a list of 13 attributes, five related to the position itself and eight linked to the neighbouring positions. Those 13 attributes were extracted from the Standard Flowgram Format (SFF) files. To properly describe the homopolymer status (presence and exact location within a homopolymer), following annotation was used: “N” for non-homopolymer, “A” and “Z” for the first and last position in the homopolymer respectively, and for the second, third, fourth, etc. position (if any) we use “B”, “C”, “D”, etc. Since the exact microbial composition is known for the TrainingDB, we were able to know for each position whether it was an error (insertion, deletion or substitution) or not based upon the original genome sequence of the organism at hand. Principal Component Analysis showed that all proposed features were needed to explain 95% of the variation in the training data (see Additional file 3).

For training the model, several classifiers were considered (multilayer perceptron, support vector machine (SVM), decision tree, naive Bayes, nearest neighborhood, logistic regression) as implemented in WEKA [27]. In order to select the best performing classifier the training dataset was split into two subsets, subset A for training, and subset B for initial testing. For constructing these subsets, the training dataset was dereplicated and distributed over subsets A and B with a ratio 1:9 in a stratified way. Afterwards, normalization of the ratio between different error types (Insertions, Deletions, Substitutions) was applied to equally train and assess each type of error (applied on subset A and B). Within subset A the native ratio between erroneous versus non-erroneous instances was 1:40, which would bias the classifier towards the more abundant one (i.e.. non-erroneous instances). Therefore subset A was reduced to have a count of 132 for each error type as well as 700 non-erroneous instances via random selection.

As evaluation criteria for assessing the best performing classifier (tested on subset B), we used the sensitivity (i.e. the proportion of actual erroneous positions that was detected as such: true positives/(true positives + false negatives)), and specificity (i.e. the proportion of actual non-erroneous positions that was detected as such: true negatives/(true negatives + false positives)). The best performing classifier was found to be an SVM with a Pearson VII Universal Kernel (PUK) [28] and sequential minimal optimization algorithm [29], which achieved a sensitivity of 0.62 and specificity of 0.95. The relative influence of each feature was assessed using the feature weights of each of them. However, as the PUK kernel is a non-linear kernel, it does not directly allow to calculate feature weights. Therefore, the feature weights were illustrated by training a linear SVM for classification, showing again that all features were essential for the optimal performance of the classifier (see Additional file 3: Table S2). The selected classifier (SVM with PUK kernel) optimally integrating these 13 features was used as error predicting tool in the NoDe algorithm. For any position predicted by this classifier in NoDe to be erroneous, the nucleotide will be marked as such.

In the second step, a modified version of the SLP algorithm [15] as implemented via pre.cluster in mothur [18] was developed. The pre.cluster implementation merges the less redundant reads with no more than 2% mismatches with the more redundant reads (parameter setting recommended in Schloss et al. [5]). In NoDe, the pre-cluster like algorithm is proceeded by a machine learning approach identifying potentially erroneous nucleotides, and those positions are masked. Accordingly, we adapted the pre-cluster algorithm implemented in NoDe in such a way that is able to ignore those masked positions in the difference calculations of pre-cluster, which means that those positions will not lead to an increase in differences. After the clustering, the remaining masked positions (i.e. positions in reads that are not merged with a more abundant one) are converted back to their original nucleotide upon rechecking the original version of the read (pre-NoDe version). A schematic representation of this approach is given in Additional file 4.

The source code and binaries for NoDe are freely available at http://science.sckcen.be/en/Institutes/EHS/MCB/MIC/Bioinformatics/NoDe.

### Benchmarking of NoDe

The MOCK1 dataset was used to benchmark NoDe to other state-of-the-art denoising tools: Pre-cluster [18], Acacia [17], Denoiser [14] and AmpliconNoise (starting with PyroNoise followed by SeqNoise [16]). Two evaluation factors were used at this stage: the quality of those sequences in terms of error rates retained after error correction and the computational cost.

To assess the capability of each algorithm we tested them on datasets after basic and strict trimming respectively. The error rate of the MOCK1 data set after basic trimming and without performing any error correction step was found to be 0.0050, with an average read length of 509 (888,635 total number of reads). NoDe resulted in the lowest error rate (0.0012) while AmpliconNoise, SLP, Acacia and Denoiser had an error rate of 0.0019, 0.0028, 0.0040 and 0.0045 respectively (Table 1). Although different denoising algorithms processed an equal amount of data (comparable average lengths and number of sequence reads), the required computational time varied dramatically. NoDe was found to yield an almost 40-fold speed improvement over AmpliconNoise (9.5 hours for NoDe versus 370 hours for AmpliconNoise on a single Intel Xeon E5-2640 2.50 GHz CPU). On the other hand, NoDe requires more computational time compared with Acacia and Pre-cluster. However, this relatively small increase in running time over the full preprocessing pipeline is largely compensated with a significant decrease of the error rate. One sample was used for additional analysis, delivering a detailed overview of the computational costs of the different preprocessing steps (trimming, aligning, filtering, denoising, etc.). An overview is given in Additional file 5. These computational costs can be further reduced via utilizing multiple cores simultaneously, which is an option available for NoDe, AmpliconNoise, Denoiser and Pre-cluster. For Acacia this parallelization can be done manually by the end user via staring up different runs in parallel. Inherent to the mode-of-action of NoDe is the linear increase of the computational time with the number of unique reads. It should be noted that this does not mean that NoDe will increase linearly with the input data, as the number of unique reads will reach to an asymptotic value upon increasing the coverage of the sample.

**Table 1:** **Benchmarking of different denoising algorithms using the MOCK1 dataset**

**Basic trimming**	**Average length**	**Error**	**CPU cost**	**Number of seq**
Denoiser	504	0.0045	112 hr	862279
Acacia	482	0.0040	8.8hr	845513
Pre-cluster	482	0.0028	8 hr	845513
AmpliconNoise	499	0.0019	370 hr	860273
NoDe	481	0.0012	9.5 hr	845513
**Strict trimming**	**Average length**	**Error**	**CPU cost**	**Number of seq**
Denoiser	439	0.0024	96 hr	785115
Acacia	424	0.0021	7.7hr	827123
Pre-cluster	424	0.0014	7 hr	827123
AmpliconNoise	424	0.0013	312 hr	818421
NoDe	425	0.0008	8.3 hr	827123

The comparison covers the final error rate as well as the computational cost (on a single CPU - Intel Xeon E5-2640 2.50 GHz) for the analysis pipelines including all tested denoising algorithms (Acacia, Denoiser, Pre-cluster, AmpliconNoise, NoDe). Also the number of reads and average read length returned by the different algorithms is displayed.

The NoDe classifier consist of three processes: 1) extracting and preparing the data, 2) running the classifier, and 3) the masking phase. The first and last process (using Perl-scripting) required a maximum of 400 MBs RAM memory using MOCK2 samples, and this during 80% of the execution time. During the residual 20%, the classifier component using the WEKA software as implemented in JAVA, required a maximum of 1,800 MBs of RAM memory. For the other denoising tools, the maximum RAM requirement was found to be 1,600 MBs, 2300 MBs, 100 MBs and 1,600 MBs for AmpliconNoise, Denoiser, Pre-cluster and Acacia, respectively.

**Figure 1:**
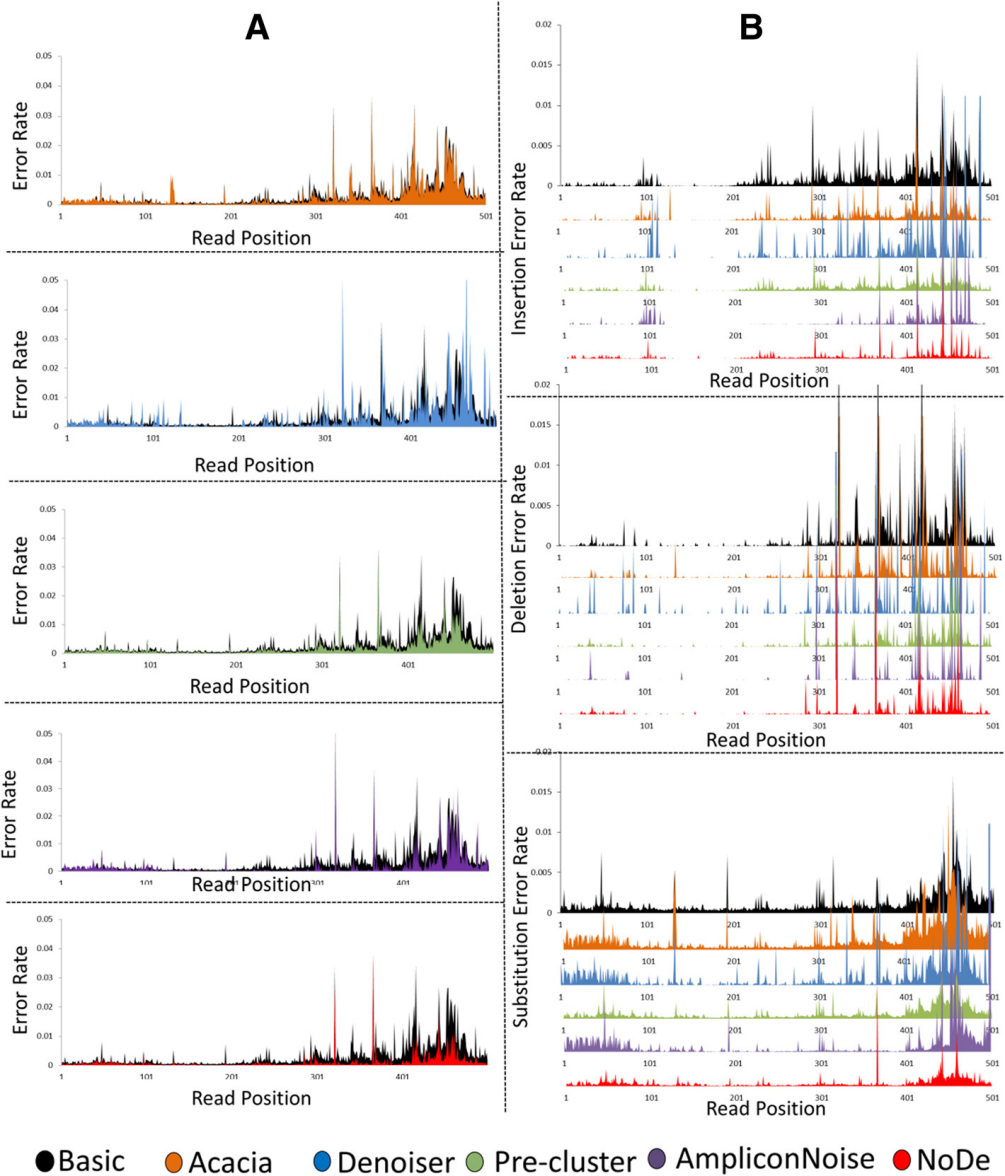
**Effect of denoising algorithms with respect to position in read. A)** Plot showing the error rate versus the position in the read after being treated with different denoising algorithms, including: Acacia (orange), Denoiser (blue), SLP (Green), AmpliconNoise (violet) and NoDe (red), with the raw error rate in black. **B)** Plots showing the insertion (upper), deletion (middle) and substitution (lower) error rates produced in the raw reads (black), as well as after being treated by different approaches, versus the position in the read.

Similarly, after applying the strict trimming step, the error rate without applying any denoising algorithm was found to be 0.0026 with an average read length of 441. Applying NoDe on these sequencing data resulted in an error rate of 0.0008, which is significantly lower than the error rates obtained with Denoiser (0.0024), Acacia (0.0021), Pre-cluster (0.0014) and AmpliconNoise (0.0013) (Table 1). A graphical representation of the effect of the different denoising tools on the sequencing data with respect to the position of the error in the read is given in Figure 1. As expected, these plots show that the total error rate (i.e. sum of the fraction of insertions, deletions and substitutions) is mainly increasing towards the end of the read. In the second plot, the performance of each denoising tool on different types of errors (average numbers are given in Additional file 6) is illustrated. Also for the strictly trimmed sequencing data, NoDe outperformed the second best benchmarked tool (AmpliconNoise) in computing time, as NoDe processed the same dataset in 8.3 hours versus 312 hours for AmpliconNoise. An overview of the computational cost of each step in the preprocessing pipeline – including the different denoising algorithms – is given in Table 2.

**Table 2:** **Tabular overview of the computational cost of the different denoising algorithms**

**Denoising approach**	**SFF extraction**	**Trim reads**	**Denoising algorithm (average memory)**	**Aligning**	**Filter alignment**	**Total time**
NoDe	00:00:12	00:00:02	00:02:16 (760 MBs)	00:06:40	00:01:00	00:10:05
Pre-cluster	00:00:12	00:00:02	00:00:13 (100 MBs)	00:06:40	00:01:00	00:08:02
AmpliconNoise	00:00:12	00:00:01	08:25:17 (1,900 MBs)	00:03:40	00:01:00	08:30:27
Denoiser	00:00:12	00:00:01	00:38:17 (2,300 MBs)	00:02:30	00:01:00	00:42:00
Acacia	00:00:12	00:00:02	00:00:55 (1,600 MBs)	00:06:40	00:01:00	00:08:49

To have an idea about the computational cost for each step, the complete pipeline was subdivided in different steps to illustrate its running time, as described above. From the table, it can be observed that the computational burden added to the complete preprocessing pipeline (by integrating the NoDe algorithm) was relatively small, and it was largely compensated with a significant improvement in the error rate, that exceeded the second best performing (but computationally intensive) algorithm AmpliconNoise. For the denoising algorithms, the average amount of memory required was added.

### Impact of error correction methods on OTU clustering

The final step in amplicon sequencing-based community profiling is the clustering of reads into Operational Taxonomic Units (OTUs), which are believed to reflect a well-delineated taxonomic group. However, different proposed amplicon sequencing processing pipelines tested on artificial communities all lead to an inflation of the number of OTUs reported, often multiple times higher than the number of bacterial species present in the tested mock community [1,3,4].

A second mock community (MOCK2, in-house produced) was used to assess the influence of different denoising and preprocessing methods on the OTU distribution. As the exact concentration of each species is known in this uneven mock community, the accuracy of the OTU clustering process can be followed up (e.g. a species assigned to more than one OTU – i.e. oversplitting – or absence of specific species). This MOCK2 community consists of 17 species spread over a wide taxonomic range and sequenced in triplicate as described in the methods section. The difference in error rates between different denoising algorithms for MOCK2 showed the same trend as for MOCK 1, i.e. an error rate of 0.0021 for AmpliconNoise, 0.0037 for Denoiser, 0.0025 for Acacia, 0.0024 for Pre-cluster while the lowest error rate of 0.0009 was achieved by NoDe.

We applied the OTU clustering algorithm using UPARSE [25] as described in the Methods section. As a validation step we first applied this algorithm on the 17 reference sequences only, resulting in 17 distinct OTUs each representing a species in the MOCK2 dataset. In the ideal case, such 17 distinct OTUs would also be returned upon analyzing the 16S amplicon pyrosequencing data. To check to which extent this could achieve using any of the denoising tools, UPARSE was applied on each of the denoised sequencing data sets obtained after applying AmpliconNoise, Pre-cluster, Acacia, Denoiser and NoDe, and the number of OTUs are reported as average number over the three replicates. NoDe had the smallest number of OTUs (22 OTUs on average, which is the closest to the optimal number of 17), while AmpliconNoise, Denoiser, Pre-cluster and Acacia had 29, 24, 46 and 46 OTUs on average respectively. When omitting the error correcting algorithms (i.e. when doing OTU clustering on the data directly after basic trimming) the number of OTUs even further inflated to 58 OTUs, pointing out the importance of integrating a denoising algorithm in a 16S rRNA metagenomics pipeline.

All the OTUs obtained were evaluated qualitatively (checking the taxonomic classification of each OTU) and quantitatively (checking the redundancy of the OTUs). For the qualitative analysis, we counted the number of “correct OTUs” (classified as one of the species present in the mock community), “noisy OTUs” (classified as one of species in the mock but unclassified at the Genus, Family or/and Order level), “missed OTUs” (species present in the mock community but totally absent in the OTU classification), “over-splitted OTUs” (correct yet redundant classification), “contaminant OTUs” (classified as species which should not be in the mock community) and “other OTUs” (OTUs unclassified at the Class level or higher).

All of the different preprocessing pipelines determined the correct relative percentage for each mock species. However, from a qualitative point of view some of the species suffered from OTU oversplitting (i.e. one species is split up over different OTUs), and is observed with all error-correcting algorithms (Table 3 and Additional file 2) however at different levels. The number of over-splitted OTUs was 4, 4, 3, 24 and 23 for NoDe, AmpliconNoise, Denoiser, Pre-cluster and Acacia, respectively. Additionally, the number of unclassified OTUs (the sum of ‘noisy’ or ‘others’ OTUs) could also be used as a quality criterion, since all organisms present in MOCK2 are well-known species present in all standard reference databases (e.g. SILVA). As such this number of unclassified OTUs should be as low as possible. For this mock community 1, 7, 5, 4 and 5 unclassified OTUs were detected for NoDe, AmpliconNoise, Denoiser, Pre-cluster, and Acacia respectively. For both aberrant types of OTUs NoDe showed the lowest number compared to other tested denoising algorithms, showing the beneficial influence of an accurate error correction tool. Additionally, over-clustering was assessed by checking the number of missed OTUs. Upon checking the closely related species (two species of Staphylococcus and three of Streptococcus) within MOCK1, we could see that on average both Denoiser and AmpliconNoise suffer from a missing OTU due to over-clustering, while NoDe, Acacia an Pre-cluster do not have this problem.

**Table 3:** **OTUs produced after treating the data with different noise removal approaches**

	**Qualitative**							**Quantitative**		
**Approaches**	**Total OTUs**	**Correclt OTUs**	**Over-splitted OTUs**	**Missed OTUs**	**Noisy**	**Contaminants**	**Others**	**Approaches**	**Rare-OTUs**	**Redundant OTUs**
**NoDe**	22	17	4	0	1	0	0	**NoDe**	4	18
**AmpliconNoise**	29	16	4	1	2	1	5	**AmpliconNoise**	11	18
**Denoiser**	24	16	3	1	1	0	4	**Denosier**	7	17
**Pre-cluster**	46	17	24	0	4	0	0	**Pre-cluster**	22	24
**Acacia**	46	17	23	0	5	1	0	**Acacia**	21	25
**Non denoised**	58	17	29	0	5	1	7	**Non Denoised**	35	17

The left side of the table displays the qualitative OTU assessment and the right side displays the quantitative analysis. For the qualitative analysis, we counted the number of “correct OTUs” (classified as one of the mock species), “noisy OTUs” (classified as one of mock species but only classified until Class, Order or Family level), “missed OTUs” (number of undetected mock species), “over-splitted OTUs” (correct yet redundant classification), “contaminant OTUs” (classified as species no belonging to mock) and “other OTUs” (OTUs unclassified at the Class level or higher). In the quantitative analysis, the number of OTUs with a redundancy below 0.1% (rare OTUs) and the ones with a redundancy above 0.1% (Redundant OTUs) were counted.

In the quantitative analysis, we count the number of OTUs with a redundancy below 0.1% (rare OTUs) and the ones with a redundancy above 0.1% (redundant OTUs). If we used the number of rare OTUs as an indicator for a better error correction step (the lower the better), the noise removal step was more accurate with NoDe resulting in only 4 rare OTUs, while AmpliconNoise, Denoiser, Pre-cluster and Acacia had 11, 7, 22 and 21 rare OTUs respectively. Indeed, the number of rare OTUs is – with exception of Denoiser – proportional to the error rate produced. Although, as Denoiser is returning the highest error rate yet resulting in a low number of rare OTUs, an extra analysis was performed plotting the expected percentage of a species versus the observed percentage after OTU clustering (Additional file 7: Figure S1). From these plots it can be derived that the percentages of the different species in the MOCK communities obtained using different denoising strategies correlate better with the actual percentages with decreasing error rate. This is largely reflected by the R-square goodness of fit value derived for each denoising algorithm, resulting in the highest value for NoDe (0.978), while observing the lowest R-square value for Denoiser (0.928) showing the highest error rate. The same conclusion could be drawn when using the method as described in Bragg et al. [17] for assessing the correspondence between the theoretical and observed proportions of the species in the mock community. Also here, the average relative deviation of the observed concentration of a species after analyzing the data versus the theoretical concentration was the lowest for NoDe, while Denoiser was one of the tools with the highest deviation (Additional file 7: Figure S2).

## Discussion

New sequencing technologies have revolutionized the way microbial communities are characterized, but still suffer from amplification and sequencing artifacts. In this work we proposed a new denoising methodology NoDe which is able to significantly reduce the sequencing error rates at a low computational cost (CPU). In general our method consists of a two-step approach. First an artificial intelligence based classifier is trained to identify those positions in 454 sequencing reads having a high sequencing error probability. These positions are identified via a set of features that are able to predict less reliable sequencing regions. By marking those positions, a quality-driven clustering of reads is made possible via a modified version of the Pre-cluster command in mothur in the second step.

When comparing our algorithm with other denoising algorithms, we could show a significant improvement at the level of error rate reduction (i.e. 37% to 73% more errors that could be corrected compared with the second best performing algorithm). As such, NoDe manages to bring the error rate of 454 pyrosequencing reads to an acceptable level in MOCK1 (0.0008 with strict trimming and 0.0012 with basic trimming) while retaining a large proportion of the sequencing data. The latter is reflected in a long read length (>400 bp) which will result in a more precise taxonomic classification [30]. Moreover, when comparing the required computational resources of NoDe with the second best denoising algorithm (AmpliconNoise), more than an order of magnitude reduction in computational cost could be obtained. The computational burden added by NoDe in the complete preprocessing pipeline as implemented in mothur is very limited, and largely compensated by the improvement in the error rate (e.g. 0.0012 versus 0.0028 in MOCK1 after basic trimming, and 0.0009 versus 0.0024 in MOCK2 for NoDe and Pre-cluster respectively).

Additionally, we could show that denoising using NoDe has a beneficial effect on the number of OTUs returned after clustering, reaching almost a one-to-one relationship between the number of OTUs and the number of species that are present in our artificial community. Moreover this process could be completed at a reasonably low computational cost. Such a close correlation between the number of OTUs and the number of present bacteria could not be achieved on the studied mock community using any of the other error correcting algorithms. Moreover, also applying the UPARSE pipeline without integrating the error correction step leads to a larger deviation from the one to one relationship between OTUs and species present in the tested communities. However, it should be noted that obtaining such a one-to-one relationship with NoDe was obtained using mock communities, and caution should be taken when extrapolating those results to real biological samples.

In this work we focused on applying error correction on 16S rRNA amplicon sequencing data. However, in principle this method is applicable to all amplicon sequencing data obtained via the Roche 454 pyrosequencing technology. As a proof of concept, we successfully tested our algorithm on the sequencing data presented in Gilles et al. [12] containing control DNA fragment type I sequences as provided with 454 sequencing kits (data not shown). Similarly, the NoDe implementation is trained and benchmarked using different 454 GS-FLX titanium sequencing data set. However a highly similar approach (eventually by fine-tuning some parameters) could also work with sequencing data obtained via the recent GS-FLX+ technology, producing reads longer than 1000 bp. Moreover, the theoretical framework presented in this paper can also be applied to other sequencing platforms like Illumina HiSeq or MiSeq, an implementation which is currently under development.

## Conclusions

We have developed a new denoising algorithm NoDe that produces lower error rates compared with other existing denoising algorithms. Moreover, using the MOCK2 community we could show that error correcting algorithms are a necessary and powerful step to come to biologically relevant numbers of OTUs, which were hard to obtain without any denoising step. NoDe is able to perform this error correcting step in a computational realistic time frame, without being a bottleneck in the preprocessing pipeline.

## Additional files

Additional file 1:
**MOCK2 composition.** Table giving the targeted concentrations for the MOCK2 community, and the exact concentrations together with the number of paralogous genes in the 16S genes of the reference genomes). Additional file 2:
**Taxonomic classification of MOCK2.** Excel sheet of the detail taxonomic classification of each of the three MOCK2 replicates after being treated with the different denoising approaches, together with the taxonomic classification of the reference sequences for the same region. Additional file 3:
**Principal Component Analysis performed on the training data.** Information on the feature selection procedure, including data on the Principal Component Analysis of the training data, and the relative weight of each feature in the classifier. Additional file 4:
**NoDe algorithm workflow.** Schematic overview showing the different steps of the NoDe algorithm. Additional file 5:
**Schematic overview of the computational cost.** Schematic overview of the computational cost of the different denoising algorithms. Additional file 6:
**Illustration of the rates of different error types.** Illustration of the percentage of different error types (insertion, deletion and substitution) after being treated by different denoising algorithms using the MOCK1 dataset. Additional file 7:
**Denoising algorithms effect on OTU level.** Three figures. A) the logarithmic percentage of each OTU against the expected percentage, B) the relative deviation from the expected value for each OTU. C) Information on extra analyses performed to assess the effect of denoising algorithms on the OTU-clustering.
