# Supplementary material for: NoDe: a fast error-correction algorithm for pyrosequencing amplicon reads
Source: BMC Bioinformatics. 2015 Mar 15;16(1):88. doi: 10.1186/s12859-015-0520-5 (PMC4403973; doi:10.1186/s12859-015-0520-5)
Supplement: Additional file 1: — MOCK2 composition. Table giving the targeted concentrations for the MOCK2 community, and the exact concentrations together with the number of paralogous genes in the 16S genes of the reference genomes). [file 12859_2015_520_MOESM1_ESM.pdf]

Additional File 1 [Table I]: Composition of the MOCK2 community with uneven concentrations. Theoretical concentrations are the concentrations as intended to be added to the mock community. Detected concentrations are obtained by aligning all reads with the reference sequences known to be present in the MOCK2 community. The table illustrates the number of paralogous 16S operons within each species with either no differences (identical paralogs), one nucleotide difference, or three nucleotide difference.

Species	Theoretical concentration (%)	Detected concentration (%)	*# Identical Paralogs*	*# Paralogs with single differences*	*# Paralogs with three differences*
Acidovorax defluvii	50	55.8	3	0	0
Pseudomonas xanthomarina	13	12.3	4	0	0
Paracoccus denitrificans	8	8.3	3	0	0
Rhodospirillum rubrum	8	5.9	4	0	0
Pseudomonas aeruginosa	4	4.1	4	0	0
Microbacterium phyllosphaerae	4	3.4	2	0	0
Arthrobacter oryzae	4	3.0	5	0	0
Delftia tsuruhatensis	1	1.2	5	0	0
Nitrosomonas europaea	1	1.1	1	0	0
Cupriavidus metallidurans	1	1.0	4	0	0
Clostridium botulinum	1	0.9	5	3	1
Staphylococcus aureus	1	0.8	3	2	0
Arthrospira platensis	0.5	0.5	2	0	0
Bacillus cereus	0.5	0.5	12	1	0
Enterococcus faecium	0.5	0.5	3	2	1
Yersinia enterocolitica	0.5	0.4	7	0	0
Desulfovibrio oxamicus	2	0.3	4	0	0
