# Supplementary material for: NoDe: a fast error-correction algorithm for pyrosequencing amplicon reads
Source: BMC Bioinformatics. 2015 Mar 15;16(1):88. doi: 10.1186/s12859-015-0520-5 (PMC4403973; doi:10.1186/s12859-015-0520-5)
Supplement: Additional file 3: — Principal Component Analysis performed on the training data. Information on the feature selection procedure, including data on the Principal Component Analysis of the training data, and the relative weight of each feature in the classifier. [file 12859_2015_520_MOESM3_ESM.pdf]

Additional File 2 [Plot I]: Illustration of principle component analysis showing biplots for component 1 versus component 2 (top left), component 1 versus component 3 (top right), component 2 versus component 3 (bottom) Bases on these plots it can be derived that for a significant portion of the data it will be hard to separate erroneous (N) and non-erroneous (T) instances.

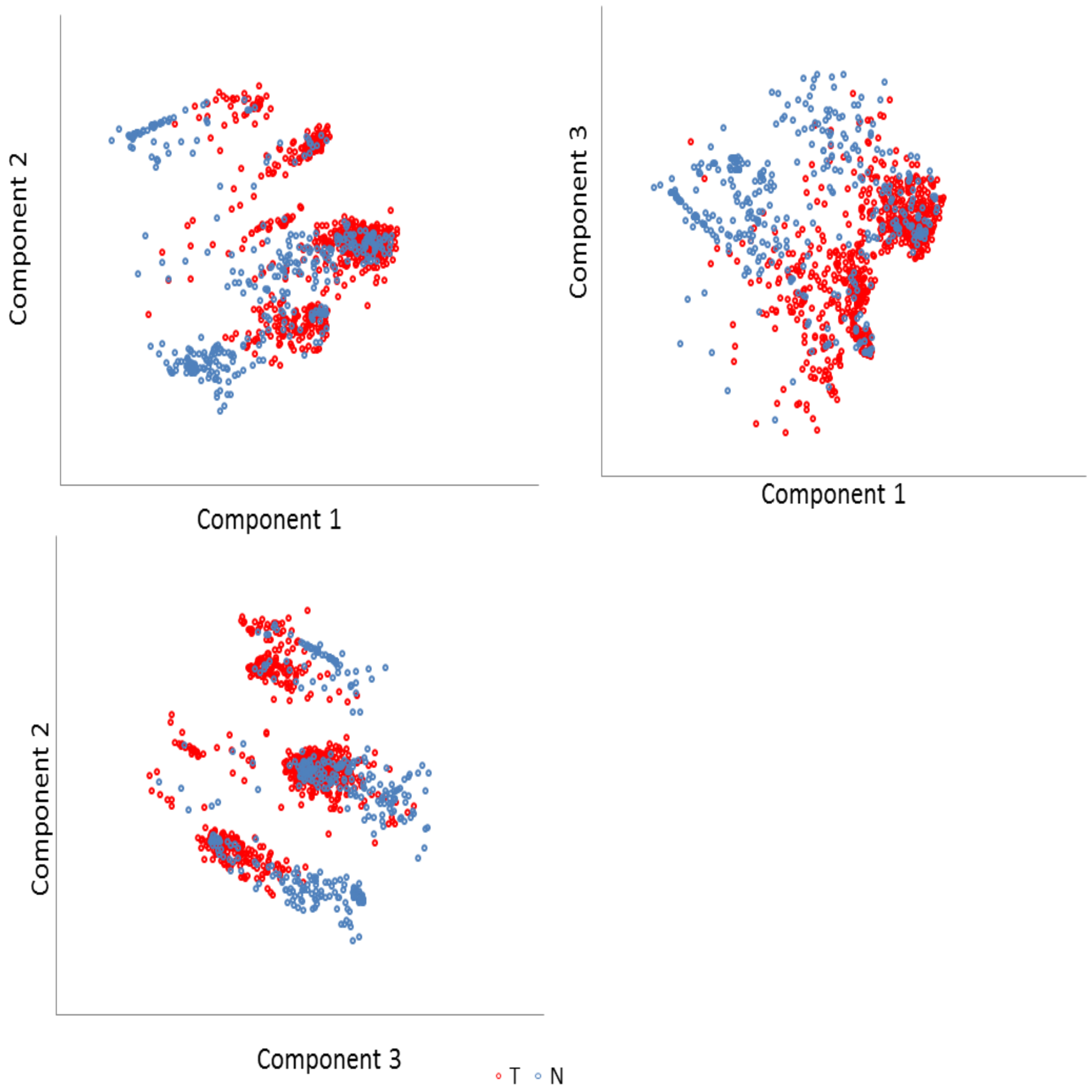

Additional File 2 [Table I]: Proportion of the variations explained by each component (highlighted in bold, second row) for the 21 components explaining 95% of the variation in the data. In addition, the relative influence of each attribute to the corresponding components, where the attributes influencing the most coloured in red.

First 11 Components	Definition	C1	C2	C3	C4	C5	C6	C7	C8	C9	C10	C11
**Proportion**		**0.159**	**0.109**	**0.087**	**0.060**	**0.057**	**0.056**	**0.051**	**0.044**	**0.038**	**0.034**	**0.031**
Position	-	-0.17	-0.08	0.30	0.03	-0.09	0.04	-0.01	0.26	-0.03	0.18	-0.02
Pre_pre_flow	before/after Noise Flowgram	0.15	0.14	0.22	0.00	-0.04	0.02	0.04	0.04	-0.05	0.00	0.03
Pos_pos_flow		0.09	-0.22	0.10	-0.18	-0.11	-0.05	-0.08	0.05	-0.14	0.17	0.13
Pre_flow	Flowgram value and Phred score of the previous, current and following position.	0.19	0.08	0.07	0.30	0.14	0.04	-0.02	0.21	-0.07	0.18	0.05
flow		0.32	-0.10	0.05	0.23	0.11	0.03	0.06	0.07	-0.02	0.03	0.05
Pos_flow		0.26	-0.30	0.01	0.17	0.12	0.05	-0.03	0.05	0.01	-0.04	0.00
Phred_before		0.35	0.12	-0.27	-0.02	0.02	-0.04	-0.06	-0.15	0.01	-0.05	0.01
Phred		0.35	0.14	-0.27	-0.06	-0.01	-0.05	-0.05	-0.18	0.03	0.03	0.01
Phred_after		0.34	0.11	-0.27	-0.09	0.00	-0.06	-0.08	-0.16	0.03	0.04	-0.01
PreHomo=N	Homopolymer status of the previous position	0.27	0.17	0.14	0.21	0.19	0.02	-0.01	0.28	-0.01	0.03	-0.07
PreHomo=A		-0.06	-0.22	-0.41	0.08	-0.22	0.03	0.01	-0.02	-0.04	-0.01	0.00
PreHomo=B		-0.10	-0.15	-0.06	-0.26	0.37	0.06	0.10	0.09	-0.02	-0.01	-0.04
PreHomo=C		-0.09	-0.16	0.08	0.12	0.07	-0.28	-0.08	0.05	-0.22	0.23	0.17
PreHomo=D		-0.12	-0.13	0.10	0.24	0.09	0.13	-0.05	-0.13	0.51	-0.18	0.02
PreHomo=E		-0.01	-0.03	0.00	0.04	0.02	0.02	-0.03	-0.14	-0.12	0.08	0.14
PreHomo=F		-0.02	-0.02	0.02	0.03	0.01	0.02	-0.01	-0.06	-0.08	0.00	-0.94
PreHomo=Z		-0.12	0.22	0.18	-0.31	-0.29	-0.01	-0.01	-0.31	-0.02	-0.06	0.08
Homo=N	Homopolymer status of the current position	0.31	0.00	0.34	-0.12	-0.14	0.01	0.18	-0.07	0.04	-0.01	-0.01
Homo=A		-0.17	0.43	-0.05	0.08	0.09	0.00	-0.23	0.10	-0.07	-0.01	0.01
Homo=B		-0.06	0.02	-0.25	0.20	-0.27	0.06	0.47	0.15	-0.01	0.04	0.02
Homo=C		-0.05	0.01	-0.06	-0.22	0.47	0.07	0.35	-0.05	-0.03	0.03	0.04
Homo=D		-0.04	-0.01	0.02	0.07	0.06	-0.66	0.12	-0.05	0.08	-0.06	-0.03
Homo=E		-0.03	0.00	0.02	0.09	0.04	0.05	-0.01	-0.04	0.67	-0.05	0.06
Homo=Z		-0.15	-0.40	-0.21	0.02	0.07	0.01	-0.27	-0.05	-0.04	0.01	-0.01
PostHomo=N	Homopolymer status of the following position	0.19	-0.22	-0.01	-0.39	-0.13	-0.08	-0.12	0.36	0.18	-0.03	-0.04
PostHomo=A		-0.01	-0.16	0.19	0.33	0.06	0.11	0.06	-0.56	-0.21	0.03	0.02
PostHomo=B		-0.05	0.16	0.01	0.09	0.09	0.01	-0.18	0.16	-0.24	-0.74	0.08
PostHomo=C		-0.05	-0.02	-0.16	0.15	-0.27	0.06	0.47	0.18	-0.06	-0.16	0.03
PostHomo=D		-0.04	-0.01	-0.03	-0.22	0.39	0.06	0.34	-0.06	-0.06	-0.08	0.05
PostHomo=E		-0.03	-0.01	0.03	0.08	0.06	-0.64	0.12	-0.04	0.08	-0.09	-0.04
PostHomo=Z		-0.17	0.37	-0.14	0.06	0.07	0.00	-0.01	0.03	0.14	0.44	-0.02
CFE	Carry forward event status	0.14	-0.01	0.27	-0.11	-0.14	0.02	0.19	-0.09	0.04	-0.06	-0.05

**Last 10 Components**	**Definition**	**C12**	**C13**	**C14**	**C15**	**C16**	**C17**	**C18**	**C19**	**C20**	**C21**
**Proportion**		**0.031**	**0.029**	**0.027**	**0.026**	**0.025**	**0.023**	**0.022**	**0.018**	**0.015**	**0.014**
Position	-	-0.08	-0.05	-0.34	-0.12	0.18	-0.22	-0.10	0.12	-0.34	-0.51
Pre_pre_flow	Flowgram of Noise before and after.	0.01	0.07	-0.26	-0.56	-0.05	0.50	-0.38	0.13	0.22	0.18
Pos_pos_flow		0.05	0.30	0.12	-0.44	0.27	-0.21	0.29	-0.53	0.17	0.02
Pre_flow	Flowgram value and Phred score of the previous, current and	0.07	0.15	-0.36	0.21	-0.27	-0.28	0.19	0.05	0.43	0.09
flow		0.07	0.14	-0.22	0.06	-0.28	-0.06	-0.10	-0.19	-0.10	-0.14
Pos_flow		0.00	-0.06	-0.03	-0.10	-0.11	0.10	-0.18	-0.18	-0.29	-0.11
Phred_before		0.02	0.02	0.01	-0.05	0.04	-0.03	-0.04	0.00	-0.06	-0.17
Phred		0.03	0.02	-0.01	-0.05	0.02	-0.03	0.02	0.03	-0.04	-0.20
Phred_after		-0.05	0.05	0.06	-0.03	-0.01	0.02	-0.03	-0.01	-0.04	-0.23
PreHomo=N	Homopolymer status of the previous position	-0.10	-0.18	0.25	-0.01	0.34	0.02	0.11	0.09	-0.01	0.07
PreHomo=A		0.05	-0.03	-0.36	0.01	0.28	-0.10	-0.15	0.04	0.07	0.22
PreHomo=B		-0.07	-0.26	0.22	-0.32	-0.43	-0.26	-0.09	0.07	0.05	-0.14
PreHomo=C		0.21	0.45	0.37	0.25	-0.08	0.25	-0.23	0.06	-0.10	-0.08
PreHomo=D		0.01	-0.08	-0.06	0.05	-0.06	0.37	0.27	-0.39	0.03	-0.15
PreHomo=E		-0.94	0.18	-0.03	0.09	-0.05	0.06	-0.01	-0.04	0.01	0.04
PreHomo=F		-0.06	0.29	0.00	-0.01	-0.04	0.02	0.01	-0.07	0.01	-0.01
PreHomo=Z		0.10	0.18	-0.21	0.05	-0.33	-0.04	0.06	-0.05	-0.05	-0.12
Homo=N	Homopolymer status of the current position	0.00	-0.01	0.05	0.06	0.00	-0.03	0.20	0.16	-0.16	0.14
Homo=A		0.00	-0.01	0.02	-0.04	0.05	0.02	-0.05	-0.14	0.13	-0.21
Homo=B		-0.02	0.04	0.03	-0.06	-0.09	-0.02	-0.06	-0.19	-0.34	0.13
Homo=C		0.04	0.11	-0.12	0.04	-0.01	-0.02	-0.05	-0.18	-0.08	0.30
Homo=D		-0.04	-0.07	-0.06	-0.05	0.01	-0.02	0.02	-0.02	0.01	0.03
Homo=E		-0.04	0.46	0.14	-0.15	0.09	-0.35	-0.24	0.26	0.03	0.05
Homo=Z		0.01	-0.06	-0.07	0.00	-0.02	0.06	-0.13	0.05	0.22	-0.09
PostHomo=N	Homopolymer status of the following position	-0.06	0.02	-0.06	0.17	-0.07	0.14	0.05	0.07	0.02	0.06
PostHomo=A		0.08	-0.10	0.07	-0.13	0.07	-0.14	0.04	0.16	0.01	0.00
PostHomo=B		-0.01	0.19	-0.02	-0.03	0.01	-0.17	-0.09	-0.13	-0.15	0.07
PostHomo=C		-0.05	0.08	0.22	-0.11	-0.12	0.10	0.14	0.12	0.38	-0.35
PostHomo=D		0.04	0.23	-0.22	0.15	0.39	0.19	0.07	0.11	0.07	-0.32
PostHomo=E		-0.06	-0.11	-0.16	-0.09	0.00	-0.09	0.05	-0.03	0.04	-0.04
PostHomo=Z		0.01	-0.06	-0.01	-0.04	-0.01	0.01	-0.12	-0.23	-0.07	0.03
CFE	Carry forward event status	-0.04	-0.22	0.12	0.33	0.15	-0.18	-0.57	-0.37	0.33	-0.10

Additional File 2 [Table II]: Support Vector Machine (SVM) calculated weights for each of the features, using a linear SVM.

Weights	Features	Weights	Features
-0.0207	Pre_pre_flow	0.5009	Homo=N
0.1855	Pre_flow	0.4389	Homo=A
-0.5378	Flow	-0.4412	Homo=B
-0.2298	Pos_flow	-0.028	Homo=C
-0.1228	Pos_pos_flow	-0.7075	Homo=D
0.0491	Position	0.237	Homo=Z
-1.4922	Phred_before	0.3748	PostHomo=N
-1.2272	Phred	0.363	PostHomo=A
-0.2001	Phred_after	-0.1697	PostHomo=B
-0.0223	PreHomo=N	0.3117	PostHomo=C
0.1204	PreHomo=A	-0.2475	PostHomo=D
-0.0559	PreHomo=B	-0.7075	PostHomo=E
0.0683	PreHomo=C	0.0752	PostHomo=Z
-0.0725	PreHomo=D	0.0174	CFE
-0.0379	PreHomo=Z		
