# Supplementary material for: NoDe: a fast error-correction algorithm for pyrosequencing amplicon reads
Source: BMC Bioinformatics. 2015 Mar 15;16(1):88. doi: 10.1186/s12859-015-0520-5 (PMC4403973; doi:10.1186/s12859-015-0520-5)

Additional File 3 [Figure I]: Schematic overview showing the different steps of the NoDe algorithm.

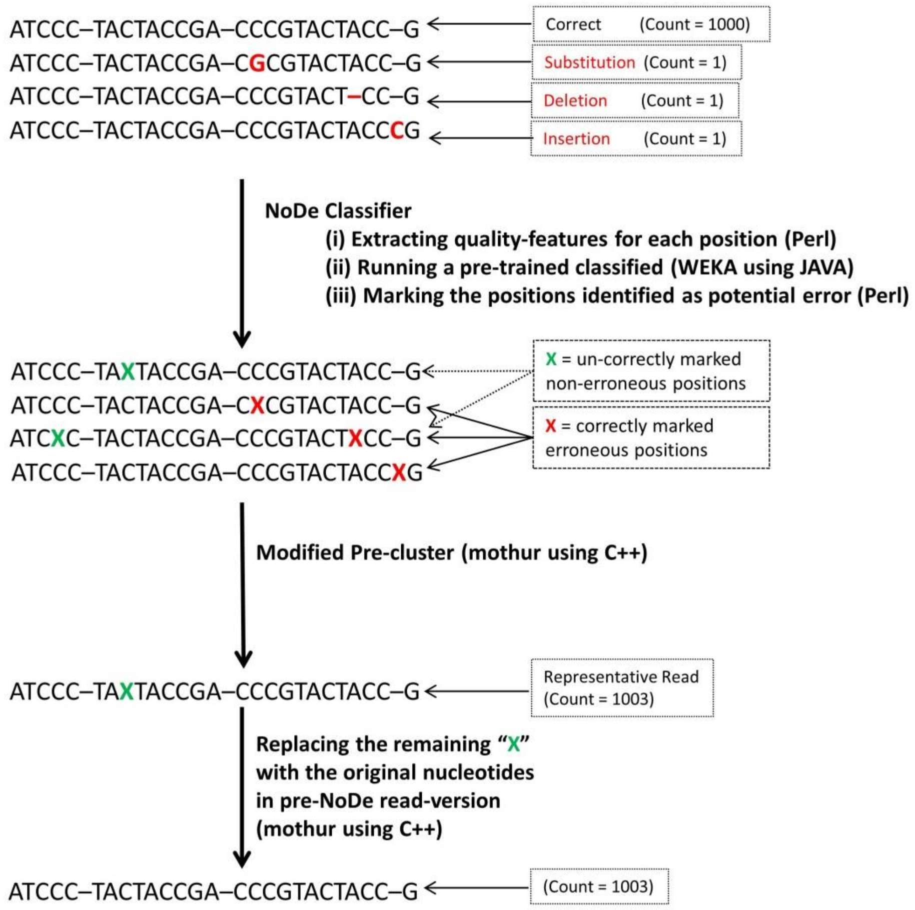

Supplement: Additional file 4: — NoDe algorithm workflow. Schematic overview showing the different steps of the NoDe algorithm. [file 12859_2015_520_MOESM4_ESM.pdf]
