# Supplementary material for: NoDe: a fast error-correction algorithm for pyrosequencing amplicon reads
Source: BMC Bioinformatics. 2015 Mar 15;16(1):88. doi: 10.1186/s12859-015-0520-5 (PMC4403973; doi:10.1186/s12859-015-0520-5)

Additional File 4 [Plot I]: Schematic overview of the computational cost of the different denoising algorithms. To have an idea about the computational cost for each step, the complete pipeline was subdivided over different steps to illustrate its running time. Trimming, aligning and filtering of the alignments are recurring steps for all approaches, as shown in the manuscript. AmpliconNoise (that consist of PyroNoise and SeqNoise) and Denoiser use as input directly the flow file (before the trimming step), but NoDe (consisting of the classifier and the modified Pre-cluster algorithm) and Pre-cluster are applied on the multiple sequence alignments. From the graph, it can be observed that the computational burden added on Pre-cluster (by integrating the NoDe algorithm) was only minor, and it was largely compensated with a significant improvement in the error rate, that exceeded the second best performing (but computationally intensive) algorithm AmpliconNoise.

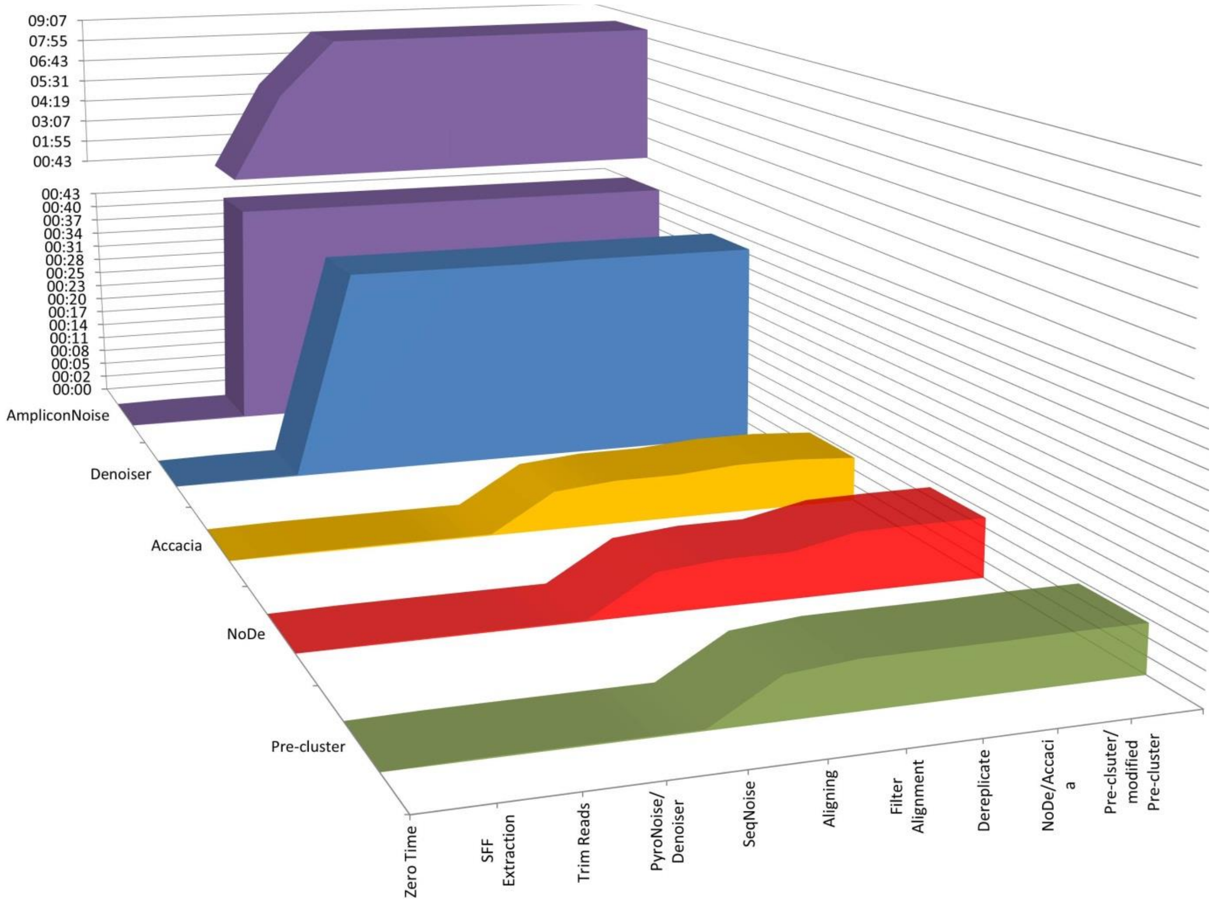

Supplement: Additional file 5: — Schematic overview of the computational cost. Schematic overview of the computational cost of the different denoising algorithms. [file 12859_2015_520_MOESM5_ESM.pdf]
