# Supplementary material for: NoDe: a fast error-correction algorithm for pyrosequencing amplicon reads
Source: BMC Bioinformatics. 2015 Mar 15;16(1):88. doi: 10.1186/s12859-015-0520-5 (PMC4403973; doi:10.1186/s12859-015-0520-5)
Supplement: Additional file 6: — Illustration of the rates of different error types. Illustration of the percentage of different error types (insertion, deletion and substitution) after being treated by different denoising algorithms using the MOCK1 dataset. [file 12859_2015_520_MOESM6_ESM.pdf]

Additional File 5 [Table I]: Illustration of the percentage of different error types (insertion, deletion and substitution) after being treated by different algorithms using the MOCK1 dataset

Basic	Insertion	Deletion	Substitution
Initial	0.0008	0.0008	0.0010
Denoiser	0.0007	0.0009	0.0009
Pre-cluster	0.0004	0.0005	0.0005
AmpliconNoise	0.0003	0.0005	0.0006
NoDe	0.0002	0.0003	0.0003
