# Supplementary material for: NoDe: a fast error-correction algorithm for pyrosequencing amplicon reads
Source: BMC Bioinformatics. 2015 Mar 15;16(1):88. doi: 10.1186/s12859-015-0520-5 (PMC4403973; doi:10.1186/s12859-015-0520-5)
Supplement: Additional file 7: — Denoising algorithms effect on OTU level. Three figures. A) the logarithmic percentage of each OTU against the expected percentage, B) the relative deviation from the expected value for each OTU. C) Information on extra analyses performed to assess the effect of denoising algorithms on the OTU-clustering. [file 12859_2015_520_MOESM7_ESM.pdf]

Additional File 7 [Figure I]: Plot with the expected percentage of each species in the MOCK2 dataset on the X-axis versus the observed percentage obtained with different denoising algorithms on the Y-axis. Percentages are converted to logarithmic scale to emphasize the low concentration species.

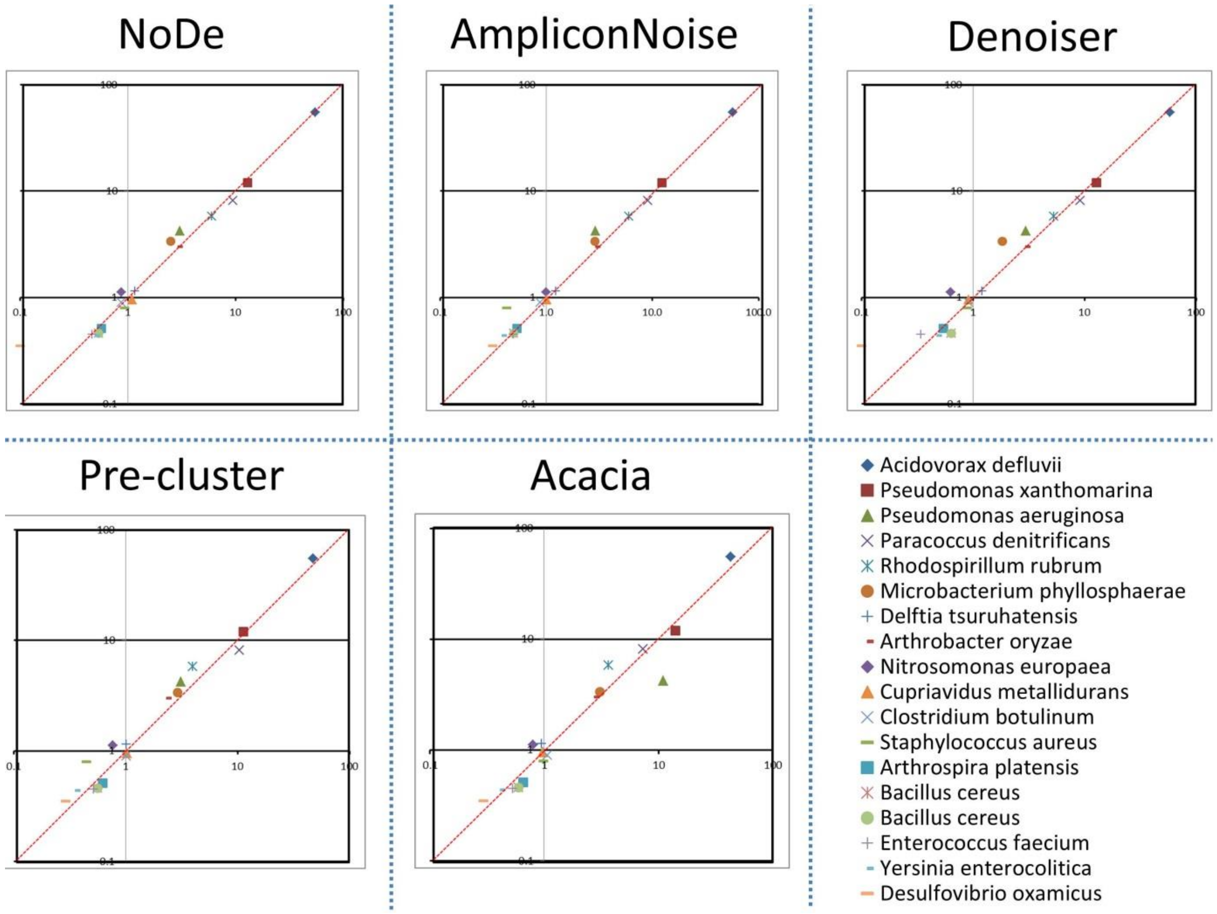

Additional File 7 [Figure II]: A plot showing the 17 OTUs of MOCK2 (in descending order of concentration) and the relative deviation of the proportion of each OTU from the true proportion after applying different denoising algorithms.

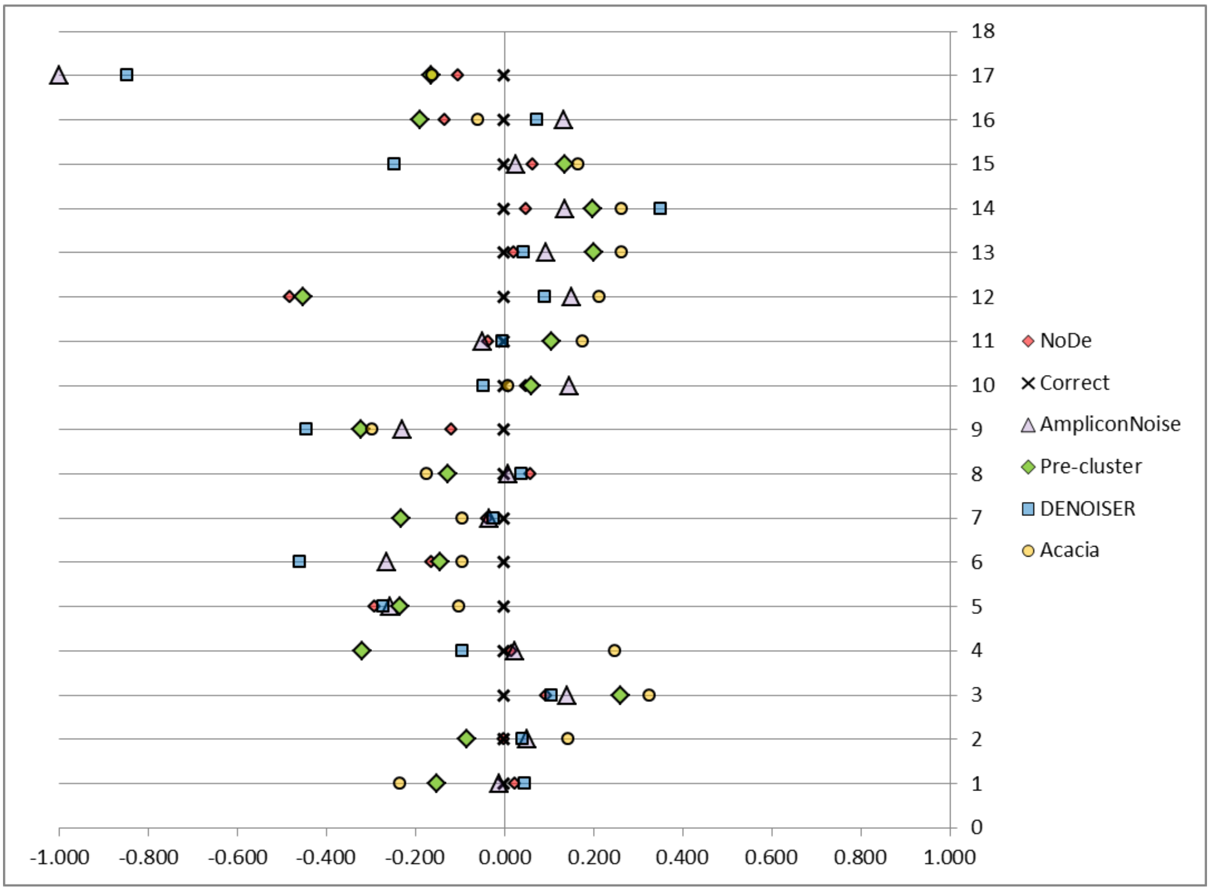

Additional File 7 [Test I]: As another method to assess over-clustering, the same test as proposed in Schloss et al. [1] was used, by applying the mothur command "sens.spec". Indeed, OTU clustering is generally performed using a 3% cutoff level, corresponding to the generally accepted level for distinguishing different species. For each pair of reads after running the denoising algorithms, we calculated the true positive (TP), false negative (FN), true negative (TN) and false positive (FP) pairs. If a pair of reads with a distance lower than 3% are merged (clustered) into one read, it is a TP; if they are not merged, it is a FN. Similarly, if a pair of reads with a distance above 3% are merged into one read, it is taken into account as a FP (indication of overclustering), while if they are not merged it is a TN (see the figure below). For this test, we used MOCK1 for the evaluation. Since we are analysing over-clustering, we will measure the specificity level (TN/(TN+FP)), as this equation contains the false positive pairs (i.e. occurrences of over-clustering). Pre-cluster, NoDe, AmpliconNoise and Acacia reported the following specificity: 1.00, 0.997, 0.914, and 0.999 respectively. It should be noted that Denoiser cannot be included in the comparative analysis, as it does not return a detailed list of IDs after running the command. This forms an obstacle tracing back the distances before running the denoising algorithms.

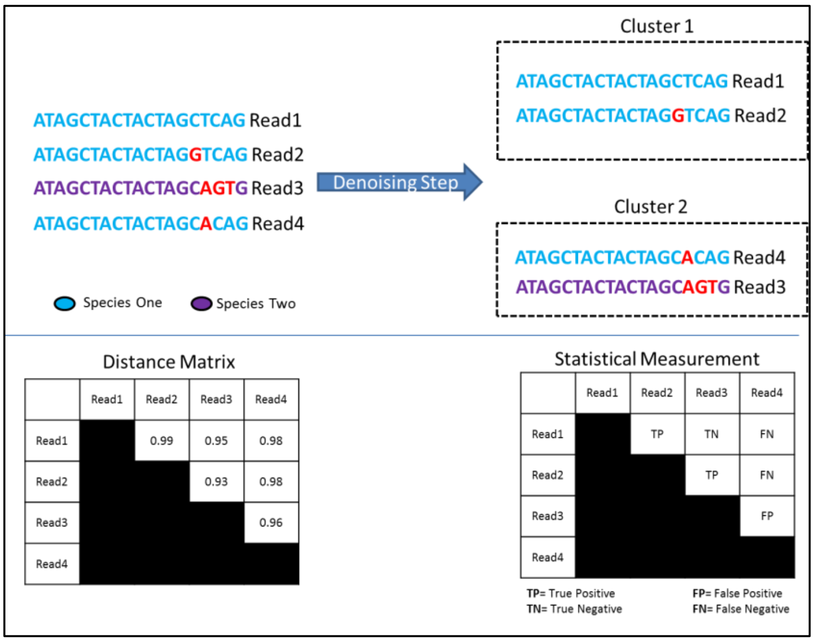

1. Schloss PD, Westcott SL: **Assessing and improving methods used in operational taxonomic unit-based approaches for 16S rRNA gene sequence analysis.** *Appl. Environ. Microbiol.* 2011, **77**:3219–26.
